# Comparative Effectiveness of Adjuvant Treatment for Resected Hepatocellular Carcinoma: A Systematic Review and Network Meta-Analysis

**DOI:** 10.3389/fonc.2021.709278

**Published:** 2021-09-02

**Authors:** Ying Liu, Yuzhu Wang, Xinkun Guo, Yifeng He, Jian Zhou, Qianzhou Lv, Xiaowu Huang, Xiaoyu Li

**Affiliations:** ^1^Department of Pharmacy, Zhongshan Hospital, Fudan University, Shanghai, China; ^2^Deparment of Hepatic Oncology, Zhongshan Hospital, Fudan University, Xiamen, China; ^3^Department of Liver Surgery and Transplantation, Liver Cancer Institute, Zhongshan Hospital, Fudan University, Shanghai, China; ^4^Key Laboratory of Carcinogenesis and Cancer Invasion, Ministry of Education, Shanghai, China

**Keywords:** hepatocellular carcinoma, adjuvant treatment, network meta-analysis, hepatic artery infusion chemotherapy, internal radiotherapy

## Abstract

**Background:**

It is controversial whether adjuvant treatment could be recommended for hepatocellular carcinoma (HCC) after curative hepatectomy. Thus, we performed a network meta-analysis (NMA) to assess adjuvant treatment’s benefit and determine the optimal adjuvant regimen.

**Methods:**

We systematically searched PubMed, Embase, and Cochrane Library for randomized controlled trials comparing adjuvant therapy *versus* no active treatment after curative hepatectomy among patients with HCC. Pooled data on recurrence and overall survival (OS) were analyzed within pairwise meta-analysis and NMA.

**Results:**

Twenty-three eligible trials (3,940 patients) reporting eight treatments were included. The direct meta-analysis showed that adjuvant therapy prevented the recurrence (OR = 0.65; 95% CI: 0.55, 0.77; P = 0.177; I^2^ = 21.7%) and contributed to OS (HR = 0.63; 95% CI: 0.54, 0.73; P = 0.087; I^2^ = 31.1%) in comparison to the observation. In the NMA, internal radiotherapy (IRT; OR = 0.55; 95% CI: 0.39, 0.77; SUCRA = 87.7%) followed by hepatic artery infusion chemotherapy (HAIC; OR = 0.6; 95% CI: 0.36, 0.97; SUCRA = 77.8%), and HAIC (HR = 0.44; 95% CI: 0.21, 0.87; SUCRA = 82.6%) followed by IRT (HR 0.54; 95% CI:0.36, 0.81; SUCRA = 69.7%) were ranked superior to other treatments in terms of preventing recurrence and providing survival benefit, respectively.

**Conclusions:**

The addition of adjuvant therapy lowers the risk of recurrence and provide survival benefit after surgical resection for HCC. HAIC and IRT are likely to be the two most effective adjuvant regimens.

**Systematic Review Registration:**

https://inplasy.com/inplasy-2020-11-0039/.

## Introduction

Liver cancer ranks as the sixth most frequently diagnosed cancer and the fourth leading cause of cancer death worldwide in 2018, with an estimated 841,000 new cases and 782,000 deaths annually (1). Hepatocellular carcinoma (HCC) is the most common primary liver cancer, which comprises 90% of cases (2). Although multiple treatments are available for patients with HCC, tumor resection performed through partial resection or liver transplantation (LT), together with ablative therapies, is proven to be the potentially curative treatment (3). Given the shortage of available organ donors, huge costs, and the restrictive criteria to select the optimal recipient, LT is not the first choice for most patients (4). And the outcomes of ablative therapies are optimized in patients with tumors smaller than 2 cm. Therefore, partial surgical resection remains the initial treatment used for early‐stage HCC (5).

Unfortunately, the 5-year recurrence rate for patients who ideally undergo surgical resection is relatively common, as high as 70% (6, 7). It has been widely accepted that recurrence occurs not because of inadequate resection, but due to the undetectable pre-existing microscopic tumor or disseminated malignant cells during operative manipulation. Recurrence of HCC generally occurs in two phases, namely, early intrahepatic metastases and late *de novo* formation of tumors (8, 9). Considering the high recurrence rate, a series of adjuvant therapies are essential to improve the prognosis of curative treatment for HCC (10, 11). Nevertheless, due to the lack of high-quality evidence, the European Association for the Study of the Liver (EASL) (12), the American Association for the Study of Liver Diseases (AASLD) (13), and the Asian Pacific Association for the Study of the Liver (APASL) (14) hold distinct recommendations on whether to take adjuvant therapies to prevent HCC recurrence. Asian guidelines generally take a positive view towards adjuvant therapies for patients with intermediate risk [single nodule >5 cm without microvascular invasion (MVI)] or high risk (single nodule >5 cm with MVI, or multiple nodules) of recurrence, while the EASL or AASLD guidelines currently do not recommend.

At present, there is no global consensus on whether the adjuvant therapies to be recommended for HCC after hepatectomy. And in the absence of direct head-to-head comparisons, the evidence proving the superiority of one adjuvant therapy over another is limited. Most published meta-analyses concerning adjuvant therapies after hepatectomy were carried out *via* traditional meta-analysis from randomized controlled trials (RCTs) and non-randomized controlled trials (NRCTs). Moreover, these studies have analyzed time-to-event outcomes using odds ratios (ORs) or relative risks (RRs) instead of hazard ratios (HRs) (15–17). A network meta-analysis (NMA) published in 2015 evaluated the efficacy of four adjuvant therapies and concluded that immunotherapy together with interferon was the most effective way to prevent recurrence, and interferon was the most efficacious therapy to prolong survival time (18). It failed to include adequate and updated trials published in the last few years, while emerging evidence of novel adjuvant therapies from RCTs is currently available. Therefore, we conducted an NMA of RCTs to compare the relative efficacy and the ranking probabilities of eight adjuvant therapies in previously curative resected patients with HCC.

## Materials and Methods

### Search Strategy and Selection Criteria

This NMA was performed in accordance with the Preferred Reporting Items for Systematic Reviews and Meta-analysis (PRISMA) extension statement for Network Meta-analysis. The method and analysis were prespecified in advance and registered on the INPLASY website (2020110039). We systematically searched (up to July 1, 2020) PubMed, Embase, and Cochrane Library. We also manually searched the relevant systematic reviews for potentially eligible articles. The searches will be refined using the Boolean term “AND” between three parts: “liver cancer,” “hepatocellular carcinoma,” “HCC,” “hepatic carcinoma,” “hepatoma”; “adjuvant,” “post-operative,” “postoperative”; “randomized controlled trial.” Studies were eligible for inclusion if they met the following criteria: (1) RCTs; (2) patients with HCC who had undergone a curative hepatectomy; and (3) reported at least one clinical outcome of interest including recurrence or overall survival (OS). The exclusion criteria were as follows: (1) duplications; (2) non-human studies; (3) NRCTs; (4) incomplete literature data; (5) review, meta-analysis, comment, and case; (6) trials not related to HCC; (7) patients with HCC who had undergone curative treatment with LT or ablative therapies; (8) adjuvant treatment with nucleos(t)ide analogues; or (9) studies focusing on irrelevant purpose.

Two authors (XG and YH) independently reviewed the titles and abstracts of selected studies, and any discrepancies were resolved through consensus with a third reviewer (YL). Full-text articles of potentially eligible studies were retrieved for further evaluation.

### Data Extraction and Quality Assessment

Two reviewers (YL and YW) independently extracted the data from the eligible studies. The following data were collected: (1) characteristics of studies and patients (authors, publication year, details of treatment, sample size, sex, age, number of tumors, tumor size, Child-Pugh score, liver cirrhosis, virology, vascular invasion and Edmondson's grading); (2) statistics for meta-analysis [the number of recurrence in each treatment arm, the HR with 95% confidence interval (CI) for OS]. Seven items specifically developed from the Cochrane risk of bias tools were used by two reviewers (YL and YW) to assess the quality of the eligible studies. Any discrepancies in data extraction and quality assessment were resolved by discussion in the whole study groups.

### Data Synthesis and Analysis

We synthesized all direct and indirect evidence to compare different treatments in terms of efficacy, reported as OR for recurrence and HR for OS, along with corresponding 95% CI. A combined OR<1 or HR<1 implied preferable efficacy in the intervention group. And it was considered statistically significant if 95% CI for the combined OR or HR did not overlap 1.

First, a traditional pairwise meta-analysis that directly compared interventions with observation were performed using STATA (version 15.0). The statistical heterogeneity in each pairwise comparison was evaluated using *I*^2^ statistic with p values. A random-effect model was used. Secondly, we used STATA (version 15.0) to generate the network meta diagram, in which edges and nodes revealed the head-to-head comparisons among interventions. The widths of edges were proportional to the number of studies comparing the two treatments. The sizes of the nodes were also proportional to the number of arms in the included studies that corresponded to the treatment. Thirdly, the NMA was conducted in the Bayesian framework with the statistical software R (version 3.6.2) and the R package “gemtc.” Both random-effect model and fixed-effect model were performed, and the best was selected based on deviance information criteria (DIC). To assess recurrence and OS, 100,000 iterations per chain (four chains, 400,000 in total) were generated with 50,000 burn-ins and a thinning interval of 1. The convergence of iterations was assessed by the Brooks-Gelman-Rubin statistic and trace plots. Global and local inconsistencies in the network were not assessed due to lacking closed loops. Within the Bayesian approach, the probability of each intervention being the most effective treatment was calculated by surface under the cumulative ranking curve (SUCRA). For each outcome, the greater the SUCRA value, the better the rank of a certain therapy among the various treatment. In addition, publication bias was evaluated *via* observing the symmetry characteristics of funnel plots and the p-value of Egger test using the package “netmeta” in software R (version 3.6.2). The symmetrical and concentrated distribution of the dots indicates no obvious deviation.

## Results

### Characteristics of Included Studies and Bias Assessment

**Figure 1:**
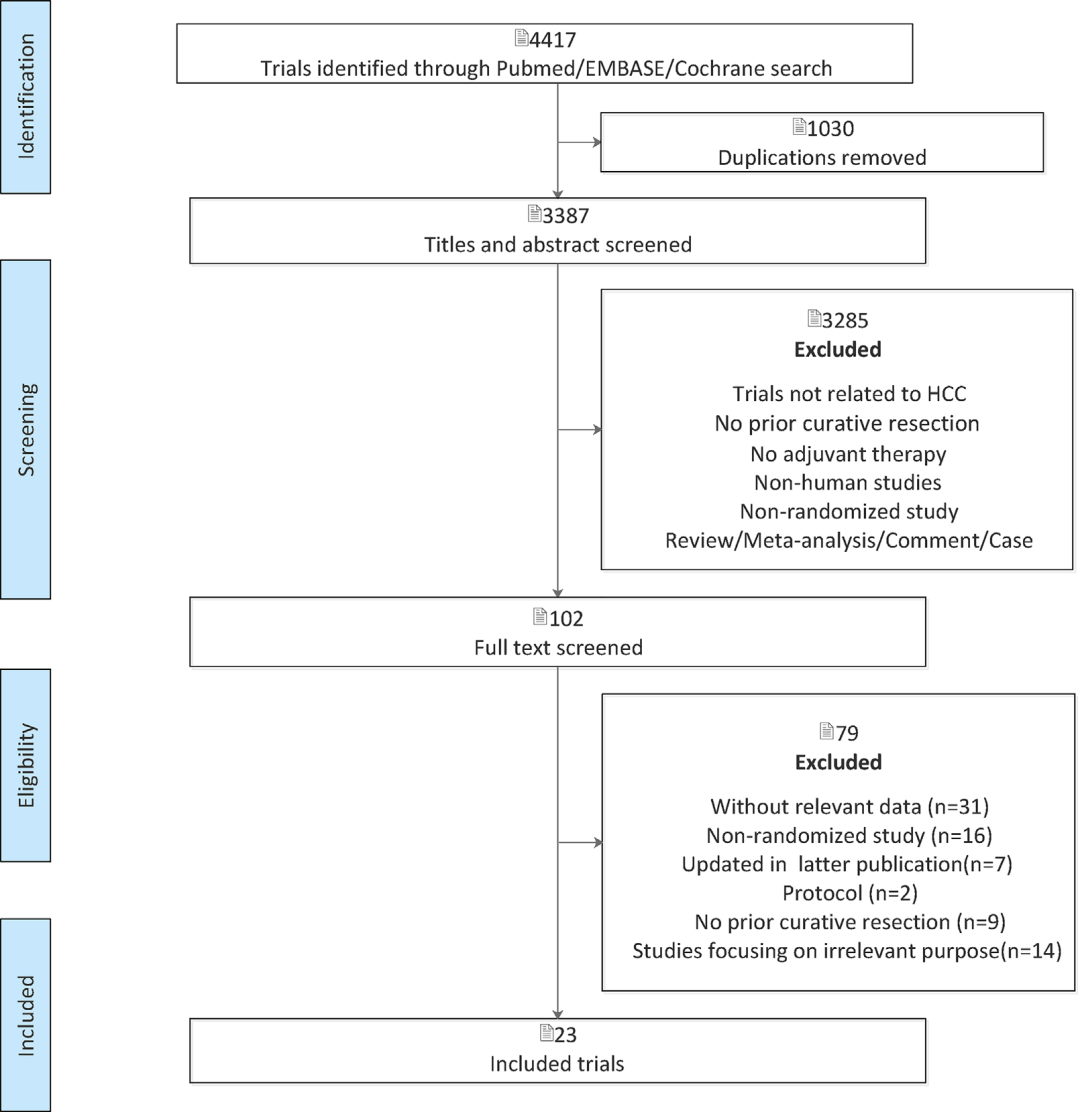
PRISMA (Preferred Reporting Items for Systematic Reviews and Meta-Analyses) flow diagram.

**Figure 2:**
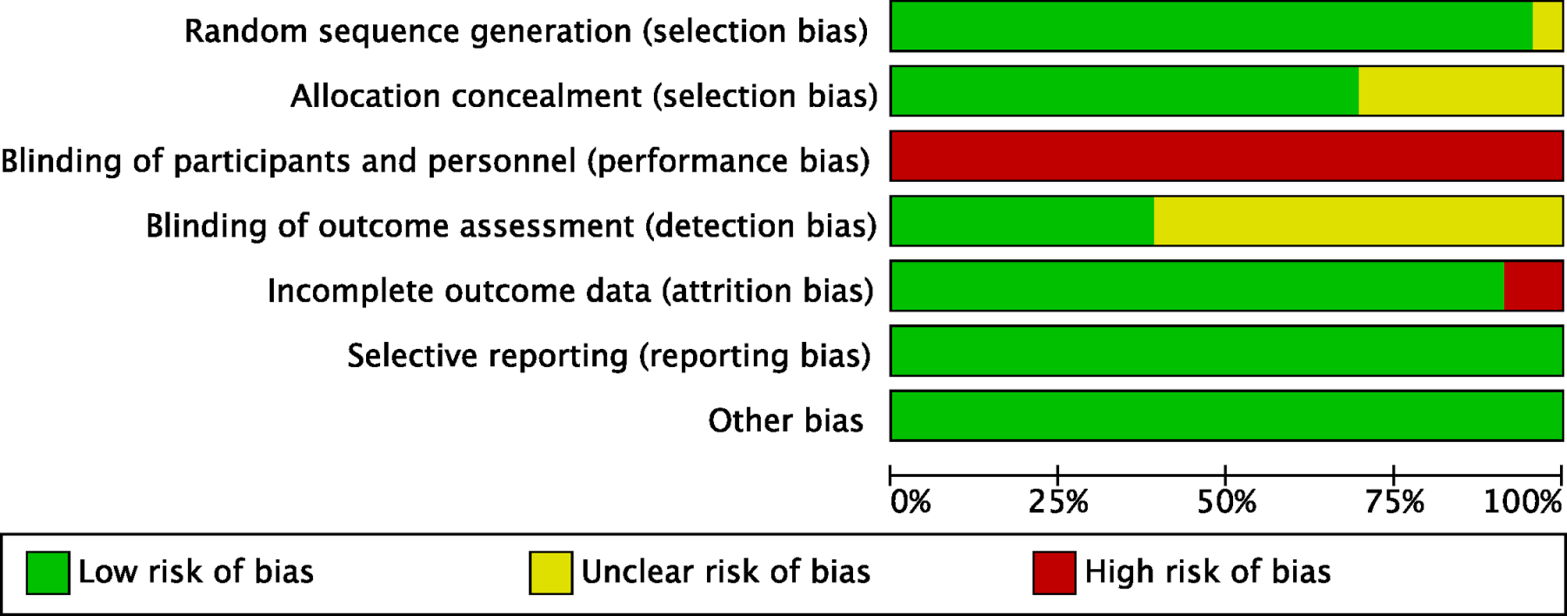
Quality assessment of included studies using Cochrane Collaboration’s Risk of Bias.

After the initial search, 4,417 relevant records were identified, of which 102 potentially eligible articles were evaluated in full text (**Figure 1**). The baseline characteristics of included studies are reported in **Table 1**. Finally, 23 RCTs met the inclusion criteria with a total of 3,940 patients, among whom 2,171 patients were enrolled to receive eight different adjuvant treatments after curative surgery and 1,769 patients were treated with surgery alone. Among the included studies, patients treated with adoptive immunotherapy (AIT) in three trials, external radiotherapy (ERT) in one trial, hepatic artery infusion chemotherapy (HAIC) in two trials, Huaier in one trial, interferon (IFN) in five trials, internal radiotherapy (IRT) in four trials, oral chemotherapy (OCT) in three trials, and transarterial chemoembolization (TACE) in four trials. The risk-of-bias assessment was performed and outlined in **Figure 2** and **Supplementary Figure 1**. All the studies included were randomized, and the trial quality was generally high, with most studies evaluated as having a low risk of bias overall. However, blinding of participants and personnel were considered impractical because of the differences between the treatment methods or almost common adverse effect. And unclear assessments were common because several articles only stated randomization and allocation concealment without detailed methods. And it is not clear whether the participants who assessed the outcomes were blind.

**Table 1:** The baseline characteristics of included studies.

Study	Year	Treatment	Sample size (n)	Sex, M/F (n)	Age (year)	Number of tumors, 1/≥2 (n)	Tumor size (cm)	Liver cirrhosis (n)	Virology, HBV/HCV (n)	Child-Pugh, A/B (n)	Vascular invasion (n)	Edmondson’s grading, I–II/III–IV (n)
Hui (19)	2009	AIT	84	63/21	NA	NA	NA	68	65/NA	68/16	36	NA
		Observation	43	34/9	NA	NA	NA	33	31/NA	34/9	23	NA
Takayama (20)	2000	AIT	76	NA	NA	51/25	NA	NA	15/50	54/22	NA	62/14
		Observation	74	NA	NA	53/21	NA	NA	14/49	50/24	NA	61/13
Xu (21)	2016	AIT	100	92/8	43 (38–56)	95/5	NA	55	84/NA	NA	2*	NA
		Observation	100	89/11	52 (43–60)	94/6	NA	58	87/NA	NA	1*	NA
Yu (22)	2014	ERT	58	51/7	53.1 ± 10.5	52/6	4.7 ± 2.6	51	53/1	NA	7	NA
		Observation	61	48/13	55.5 ± 10.7	53/8	5.6 ± 3.7	54	53/5	NA	8	NA
Huang (23)	2015	HAIC	42	31/11	59.1 ± 6.2	24/18	6.2 ± 1.5	NA	NA	24/18	NA	NA
		Observation	43	30/13	58.4 ± 5.7	23/20	5.7 ± 1.3	NA	NA	27/16	NA	NA
Li SH (24)	2020	HAIC	58	52/6	54 (25-69)	36/22	NA	32	54/2	NA	NA	23/35
		Observation	58	49/9	55.6 ± 1.6	42/16	NA	35	51/1	NA	NA	29/29
Chen (25)	2018	Huaier	686	565/121	NA	595/91	NA	473	544/8	643/43	NA	488/198
		Observation	316	255/61	NA	274/42	NA	198	234/5	291/25	NA	223/93
Chen (26)	2012	IFN	133	108/25	50 (48–54)	103/30	3.5 (3.04.0)	73	106/27	NA	41*	NA
		Observation	135	112/23	49 (46–51)	115/20	3.0 (2.5–3.5)	74	108/26	NA	33*	NA
Lo (27)	2007	IFN	40	31/9	49 (26–75)	33/7	5.5 (1.8–22)	19	38/1	NA	NA	NA
		Observation	40	34/6	54 (24–74)	29/11	5.7 (1.2–18)	19	39/2	NA	NA	NA
Mazzaferro (28)	2006	IFN	76	61/15	65 (41–74)	NA	NA	NA	NA	70/6	NA	NA
		Observation	74	51/23	67 (36–73)	NA	NA	NA	NA	70/4	NA	NA
Nishiguchi (29	2005	IFN	15	15/0	61.9 ± 5.8	NA	2.5 (1.9–3.5)	NA	NA	11/4	NA	10/5
		Observation	15	15/0	60.0 ± 4.8	NA	2.6 (2.4–3.5)	NA	NA	12/3	NA	8/7
Sun (30)	2006	IFN	118	106/12	52.2	102/16	4.3 ± 2.7	98	NA	NA	90*	86/32
		Observation	118	102/16	50.4	103/15	4.9 ± 3.0	104	NA	NA	89*	77/41
Chen (31)	2013	IRT	34	25/9	50.8 ± 6.8	30/4	6.24 ± 2.55	18	26/6	34/0	17*	22/12
		Observation	34	24/10	48.9 ± 7.3	31/3	5.65 ± 2.52	20	31/5	34/0	14*	23/11
Chung (32)	2013	IRT	51	41/10	65 (22–82)	NA	4.2 (0.4–30)	NA	29/NA	NA	NA	34/16
		Observation	52	45/7	63 (42–84)	NA	3.8 (1.4–18)	NA	32/NA	NA	NA	32/20
Lau (33)	1999	IRT	21	17/4	51 (23–71)	14/7	4.4 (1.4–11)	NA	19/NA	NA	1	17/2
		Observation	22	18/4	54 (24–75)	18/4	3.8 (1.5–10)	NA	19/NA	NA	1	18/2
Li J (34)	2020	IRT	78	58/20	53 (47–59)	73/5	4.9 (3.2–6.4)	42	66/0	NA	31	29/49
		Observation	78	61/17	53 (47–58)	74/4	5.3 (3.2–7.3)	45	60/3	NA	32	27/51
Hasegawa (35)	2006	OCT	79	60/19	65 (29–75)	53/26	3.3 (1.2–12)	42	14/58	68/11	18	NA
		Observation	80	65/15	64 (35–78)	58/22	3.4 (7–13)	38	15/56	70/10	17	NA
Xia (36)	2010	OCT	30	25/5	NA	25/5	7.27 ± 4.37	19	26/NA	NA	18	NA
		Observation	30	21/9	NA	26/4	6.34 ± 3.16	21	24/NA	NA	20	NA
Yamamoto (37)	1996	OCT	28	NA	NA	NA	NA	NA	NA	NA	NA	NA
		Observation	27	NA	NA	NA	NA	NA	NA	NA	NA	NA
Peng (38)	2009	TACE	51	46/5	46.2 ± 13.8	NA	9.04 ± 3.02	42	31/5	44/7	NA	NA
		Observation	53	50/3	50.2 ± 7.5	NA	8.39 ± 2.29	37	40/3	46/7	NA	NA
Wang (39)	2018	TACE	140	121/19	54.2 ± 9.7	102/38	NA	140	NA	NA	78*	81/59
		Observation	140	109/31	52.6 ± 10.3	109/31	NA	140	NA	NA	87*	80/60
Wei (40)	2018	TACE	116	106/10	44 (18-75)	NA	NA	50	94	116/0	NA	NA
		Observation	118	106/12	48.5 (18-74)	NA	NA	42	101	116/2	NA	NA
Zhong (41)	2009	TACE	57	53/4	47.6 ± 10.4	13/44	9.5 ± 3.8	NA	53/NA	56/1	NA	35/22
		Observation	58	49/9	48.2 ± 11.2	16/42	9.7 ± 3.6	NA	52/NA	58/0	NA	34/24

*Microvascular invasion.

NA, Not Available.

### Pairwise Meta-Analysis

The detailed forest plot of the results is presented in **Supplementary Figure 2** for recurrence and **Supplementary Figure 3** for OS. Original ORs with 95% CIs were reported in 22 studies (3,836 patients) for recurrence and HRs with 95% CIs in 21 studies (3,663 patients) were informed for OS. An overall OR of 0.65 (95% CI, 0.55, 0.77; P = 0.177; I^2^ = 21.7%) and HR of 0.63 (95% CI, 0.54, 0.73; P = 0.087; I^2^ = 31.1%) revealed the efficacy of adjuvant group over observation group. When compared to observation, HAIC [OR 0.50 (0.28, 0.89)], Huaier [OR 0.58 (0.44, 0.76)], and IRT [OR 0.41 (0.27, 0.64)] showed significantly lower risk of recurrence, and trended toward improvements were presented in AIT [OR 0.69 (0.45, 1.07)], ERT [OR 0.68 (0.33, 1.41)], IFN [OR 0.89 (0.66, 1.19)], OCT [OR 0.60 (0.27, 1.34)], and TACE [OR 0.71 (0.45, 1.14)]. Pooled HRs strongly favored the adjuvant treatment of AIT [HR 0.64 (0.43, 0.94)], HAIC [HR 0.45 (0.25, 0.79)], Huaier [HR 0.55 (0.33, 0.92)], IFN [HR 0.61 (0.39, 0.94)], IRT [HR 0.54 (0.38, 0.79)], and TACE [HR 0.63 (0.51, 0.78)] in significantly improving OS.

### Network Meta-Analysis

**Figure 3:**
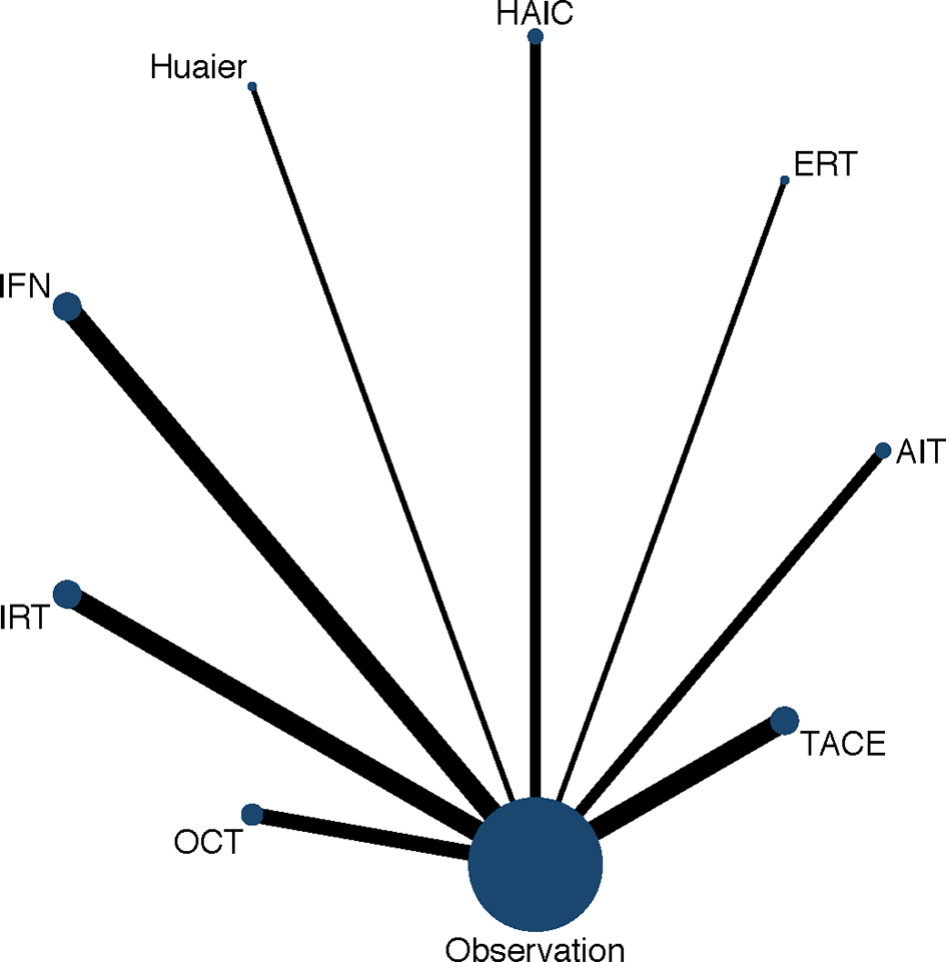
Network diagram of eligible comparisons for OS. Each circular node represents a variety of interventions. The circle size is proportional to the number of randomly assigned participants. The width of lines between the nodes is proportional to the number of trials performing head-to-head comparisons. AIT, adoptive immunotherapy; ERT, external radiotherapy; HAIC, hepatic artery infusion chemotherapy; IFN, interferon; IRT, internal radiotherapy; OCT, oral chemotherapy; TACE, transarterial chemoembolization.

**Figure 3** presents the network of eligible comparisons for OS and the network diagram for recurrence is shown in **Supplementary Figure 4**. Most of the included studies have OS and recurrence as the endpoints, except Hui 2009 and Mazzaferro 2006 have the endpoint of recurrence, while Peng 2009 used OS as the endpoint. The consistency and inconsistency models were compared using the deviance information criterion, which indicated that the data was basically consistent.

**Figure 4:**
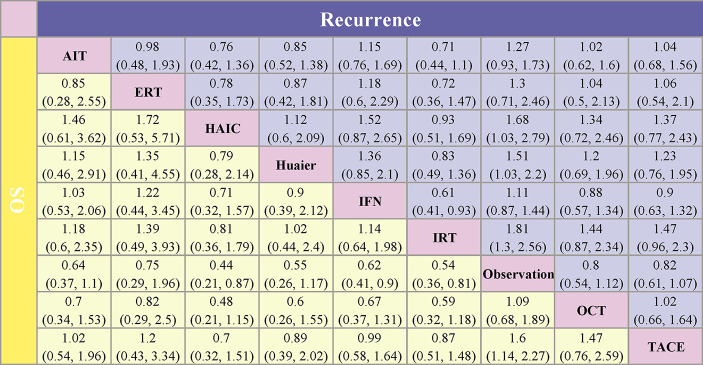
Pooled estimates of the network meta-analysis. Data in each cell are hazard or odds ratios (95% confidence intervals) for the comparison between the column defining intervention and the row defining intervention. For the lower triangle (overall survival), hazard ratios less than 1 favor the treatment in the corresponding column. For the upper triangle (recurrence), odds ratios less than 1 favor the treatment in the corresponding row. AIT, adoptive immunotherapy; ERT, external radiotherapy; HAIC, hepatic artery infusion chemotherapy; IFN, interferon; IRT, internal radiotherapy; OCT, oral chemotherapy; TACE, transarterial chemoembolization.

**Figure 5:**
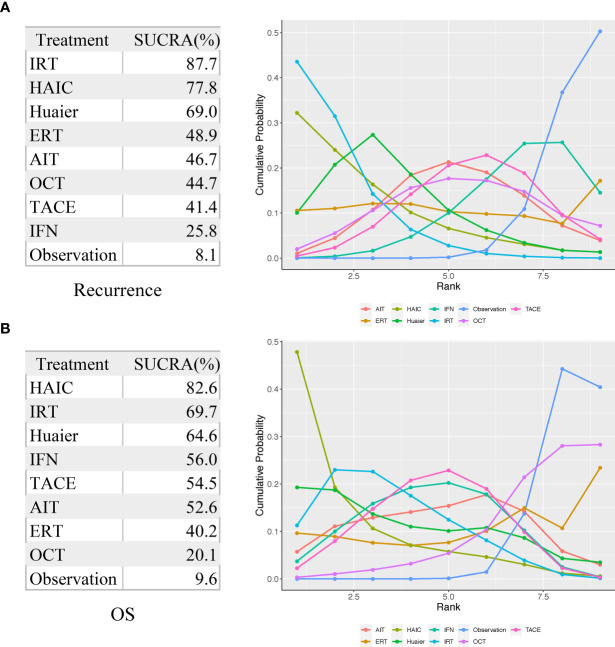
Surface under the cumulative ranking curve (SUCRA) values for recurrence **(A)** and OS **(B)**. AIT, adoptive immunotherapy; ERT, external radiotherapy; HAIC, hepatic artery infusion chemotherapy; IFN, interferon; IRT, internal radiotherapy; OCT, oral chemotherapy; TACE, transarterial chemoembolization.

As shown in **Figures 4** and **5**, indirect comparison by network meta-analysis suggested a lower risk of recurrence with the help of adjuvant therapy when compared to surgery alone group (observation group). Briefly, adjuvant treatment of IRT was ranked best in preventing recurrence [OR 0.55 (0.39, 0.77) and SUCRA = 87.7%], followed by HAIC [OR 0.6 (0.36, 0.97); SUCRA = 77.8%], Huaier [OR 0.66 (0.45, 0.97); SUCRA = 69%], ERT [OR 0.77 (0.41, 1.42); SUCRA = 48.9%], AIT [OR 0.79 (0.58, 1.07); SUCRA = 46.7%], OCT [OR 0.8 (0.54, 1.12); SUCRA = 44.7%], TACE [OR 0.82 (0.61, 1.07); SUCRA = 41.4%], and IFN [OR 0.9 (0.69, 1.14); SUCRA = 25.8%].

For improving OS, HAIC was superior to all other adjuvant treatment as compared to observation group with HR 0.44 (0.21, 0.87) and SUCRA = 82.6%, followed by IRT [HR 0.54 (0.36, 0.81); SUCRA = 69.7%], Huaier [HR 0.55 (0.26, 1.17); SUCRA = 64.6%], IFN [HR 0.62 (0.41, 0.9); SUCRA = 56%], TACE [HR 0.62 (0.44, 0.88); SUCRA = 54.5%], AIT (HR 0.64 (0.37, 1.1); SUCRA = 52.6%), ERT [HR 0.75 (0.29, 1.96); SUCRA = 40.2%], and OCT [HR 0.92 (0.53, 1.47); SUCRA = 20.1%].

### Assessment of Publication Bias

As shown in **Supplementary Figure 5**, there was no significant asymmetry among the included studies in terms of recurrence and OS. The p-value of Egger’s test for recurrence was 0.846 and for OS was 0.995. Hence, it can be concluded that there is less likelihood of publication bias.

## Discussion

Given that the benefits of adjuvant therapies compared to surgery alone after curative resection for HCC remains to be clearly defined, we combined direct and indirect evidence from 23 RCT comparing eight different adjuvant treatments with a total of 3,940 participants. The results suggested that adjuvant treatments provide survival benefits over surgery alone. HAIC and IRT probably provide the fewest recurrence and the best survival among all the post-operative therapeutic interventions evaluated, as evidenced by their SUCRA values.

Unlike systemic chemotherapy, HAIC can directly deliver chemotherapeutic drugs to the tumor supplying artery with increased local concentration, and thus achieve better inhibition of tumor recurrence and milder adverse effects, even for patients with marginal liver function (42). HAIC has attracted wide attention in Asia, especially in Japan, where HAIC is recommended as the standard therapy in the treatment of TACE-refractory patients and patients with portal branch tumor thrombus (PVTT) (43). Most published reports have observed that HAIC may reduce the risk of recurrence after hepatectomy for HCC patients with macroscopic PVTT. Patients with high-grade vascular invasion are good candidates for the adjuvant treatment of HAIC (44–46). A meta-analysis demonstrated that adjuvant HAIC improved PFS and OS after hepatectomy, especially in tumors larger than 7 cm (47). However, patients prefer TACE and oral anticancer drugs rather than HAIC due to the complexity of managing the implanted catheter system, so there is currently insufficient data of RCTs (48). And the optimal regimen for HAIC remains a controversial issue. Various regimens have been reported, including single or combined administration of cisplatin, 5-fluorouracil, oxaliplatin, doxorubicin, epirubicin, and mitomycin C (42).

During the last decade, HCC is generally considered to be a radiosensitive tumor (49). However, most international guidelines still do not recommend ERT to treat HCC with few exceptions due to the severe hepatotoxicity of the normal tissues after absorbing radiation more than 35 Gy. Since the early 1990s, radiotherapy (RT) has experienced tremendous technological advancements to develop IRT, which can precisely deliver very high tumoricidal dose to the tumor while preserving normal liver parenchyma (50). According to the pharmacokinetics of radionuclides, IRT can be properly indicated in HCC accompanied by PVTT with the OS reaching more than 20 months (51). A variety of radioisotopes, such as 131I-lipiodol for radioembolization (32, 33), 131I-metuximab for radioimmunotherapy (34), and iodine-125 for brachytherapy (31), have also been verified to be used as adjuvant therapies after curative hepatectomy. Side effects reported with IRT were generally moderate and manageable. Given the variability in radiosensitivity of the patient and the decisive role of absorption dose in the biological effects of IRT, the dosimetry of radionuclide therapy has gradually attracted much attention (52).

The strength of our research is that we used NMA to compare eight different adjuvant treatments for HCC simultaneously, while most of the previous analyses have been carried out *via* traditional meta-analysis from both RCTs and NRCTs. We excluded studies concerning nucleos(t)ide analogues, because the necessity of its administration for many years or for life in patients with HBV-related HCC has been discussed in many research (53, 54). In this NMA, the adjuvant treatments included are given for a finite duration. A previously published NMA of 14 trials by Zhu et al. in 2015 provided evidence for the superior survival benefit with the treatment of IFN. However, without adequate and updated trials, only AIT, IFN, IRT, and OCT have been taken into consideration for comparing the efficacy in the review (18). Consistent with earlier meta-analysis, results of our analysis suggested that adjuvant therapies contributed to OS, except for ERT and OCT. Contrast to some previous studies, administered with TACE and IFN showed no benefit of preventing recurrence. This result could be partly illustrated with the subgroup analysis by Huang et al. (55) and Xu et al. (56), which suggested that adjuvant IFN significantly reduces the recurrence of HCV-related HCC rather than HBV-related HCC. And some meta-analysis reported the clinical benefit of adjuvant TACE for HCC with risk factors (multiple nodules, tumors ≥5 cm or vascular invasion) (15, 57, 58).

Several limitations of this study deserve further discussion. First, though only RCTs were included, double-blind was considered impractical due to the difference between the adjuvant treatment methods or almost common adverse effect, and some eligible studies showed unclear risk of bias, especially in terms of allocation concealment and blinding of outcome assessment. Second, it is impossible to precisely integrate or incorporate data from each study for all endpoints into the analysis in the absence of original data. As a well-recognized outcome in adjuvant trials, recurrence-free survival (RFS) takes account of whether and when the event occurred. However, some trials defined RFS as the time from randomization to the first recurrence or death due to any cause, while other trials defined RFS without the endpoint of death. Hence, recurrence instead of RFS was considered as the outcome. Since many studies did not provide HRs for OS, they were estimated from the reported log-rank p values and the events in each arm according to the procedure in the study by Tierney et al. (59). Third, unavoidable confounding factors remain in this NMA, manifesting in the difference of follow-up time, post-operative staging, HBV/HCV infection, and so on. However, it was not available to perform subgroup NMA for these confounding factors with limited reporting outcomes. A further stratified analysis will help us clarify the indications of adjuvant treatments. Fourth, in the absence of sufficient direct head-to-head comparisons, most treatments were compared indirectly, and the most direct evidence came from a single trial. In addition, some new methods for adjuvant therapy without comparing in any RCTs were not included, such as multitarget tyrosine kinase inhibitors (TKIs), immune checkpoint inhibitors (ICIs), or the combination of both.

Although TKIs and ICIs are generally used in patients with advanced-stage HCC, their use after curative resection is still controversial. The phase III STORM trial was designed to compare the efficacy and safety of sorafenib as adjuvant therapy in patients who have undergone curative surgery or local ablation. Sorafenib not only failed to show superiority over placebo in terms of RFS (HR=0.940; 95% CI: 0.780, 1.134; p=0.26), but it was also accompanied by an increased grade 3 or 4 adverse events (60). It is unclear that the dose of sorafenib lower than the intended 800 mg or eligible patients with a lower risk of recurrence was a contributing factor to the negative findings reported. Except for the STORM trial, there are currently no published RCTs evaluating the efficacy of TKIs and ICIs as adjuvant therapy. NCT04227808 is an ongoing trial to evaluate the use of adjuvant lenvatinib in HCC. In CheckMate 9DX and KEYNOTE-937, nivolumab and pembrolizumab are being investigated in the adjuvant setting for patients with HCC, respectively. Numerous combination regimens for advanced HCC comprise PD-1/PD-L1 blockade plus antiangiogenic agents have demonstrated improved outcome data. In an NMA of 14 trials, the combination of atezolizumab and bevacizumab was found to be the most preferred therapy for patients with HCC compared with sorafenib (HR=0.58; 95% CI: 0.42–0.80), lenvatinib (HR=0.63; 95% CI: 0.44–0.89), and nivolumab (HR=0.68; 95% CI: 0.48–0.98) (61). Looking forward to the findings of the ongoing EMERALD-2 and IMbrave050 trials, which evaluated the combined efficacy of anti-PD-L1 antibody and VEGF antibody in adjuvant therapy.

## Conclusion

Among people with previously resected HCC, HAIC and IRT are likely to be the most two effective adjuvant treatments to prevent recurrence and improve OS. However, these adjuvant regimens have not yet undergone a direct head-to-head comparison. The final decision on adjuvant therapy requires a multidisciplinary consultation, and the potential risks and benefits should be considered to prolonging the survival of HCC. Further clinical researches are warranted to confirm or condemn our findings and to predict patients with a higher likelihood of response to adjuvant therapy.

## Data Availability Statement

The original contributions presented in the study are included in the article/**Supplementary Material**. Further inquiries can be directed to the corresponding authors.

## Author Contributions

XL and XH designed the conception and wrote the protocol. XG and YH retrieved and reviewed the literature. YL and YW extracted the data and assessed its quality. JZ and QL guided the statistical analysis. YL and XG analyzed the data. YL wrote the manuscript. All authors contributed to the article and approved the submitted version.

## Funding

This study was supported by the National Natural Science Foundation of China (No. 81803073) and Zhongshan Hospital Affiliated to Fudan University Foundation for Innovation (No. 2020ZSCX09).

## Conflict of Interest

The authors declare that the research was conducted in the absence of any commercial or financial relationships that could be construed as a potential conflict of interest.

## Publisher’s Note

All claims expressed in this article are solely those of the authors and do not necessarily represent those of their affiliated organizations, or those of the publisher, the editors and the reviewers. Any product that may be evaluated in this article, or claim that may be made by its manufacturer, is not guaranteed or endorsed by the publisher.
