# Supplementary material for: Comparative Effectiveness of Adjuvant Treatment for Resected Hepatocellular Carcinoma: A Systematic Review and Network Meta-Analysis
Source: Front Oncol. 2021 Sep 2;11:709278. doi: 10.3389/fonc.2021.709278 (PMC8445365; doi:10.3389/fonc.2021.709278)

Supplementary Figure 1. Each risk of bias item for each included study.


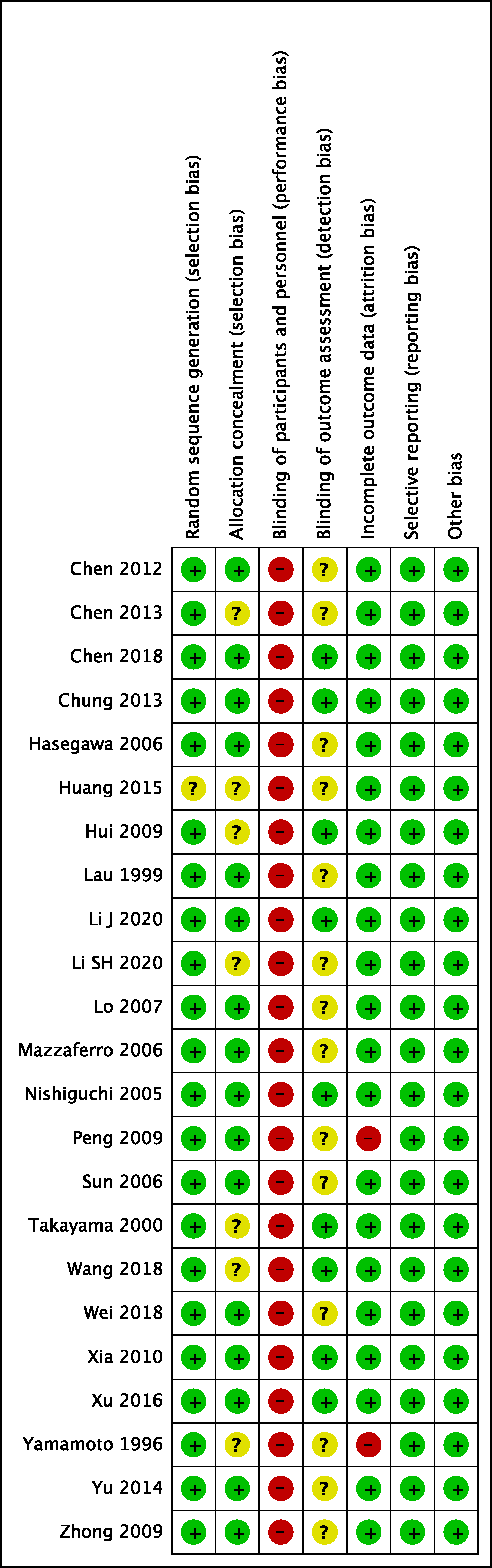


Supplementary Figure 2. Forest plot of recurrence for conventional meta-analysis.


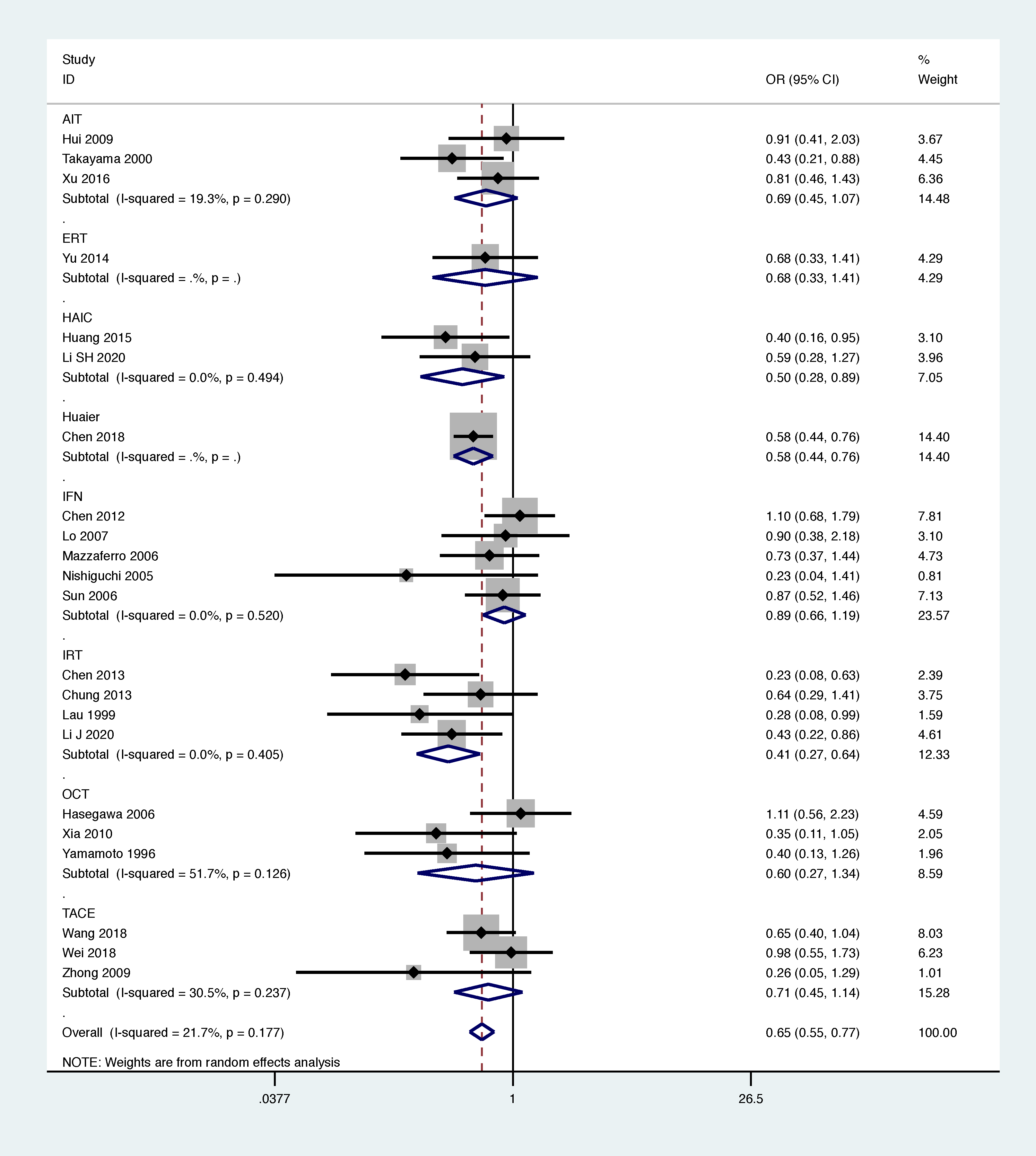


Supplementary Figure 3. Forest plot of OS for conventional meta-analysis.


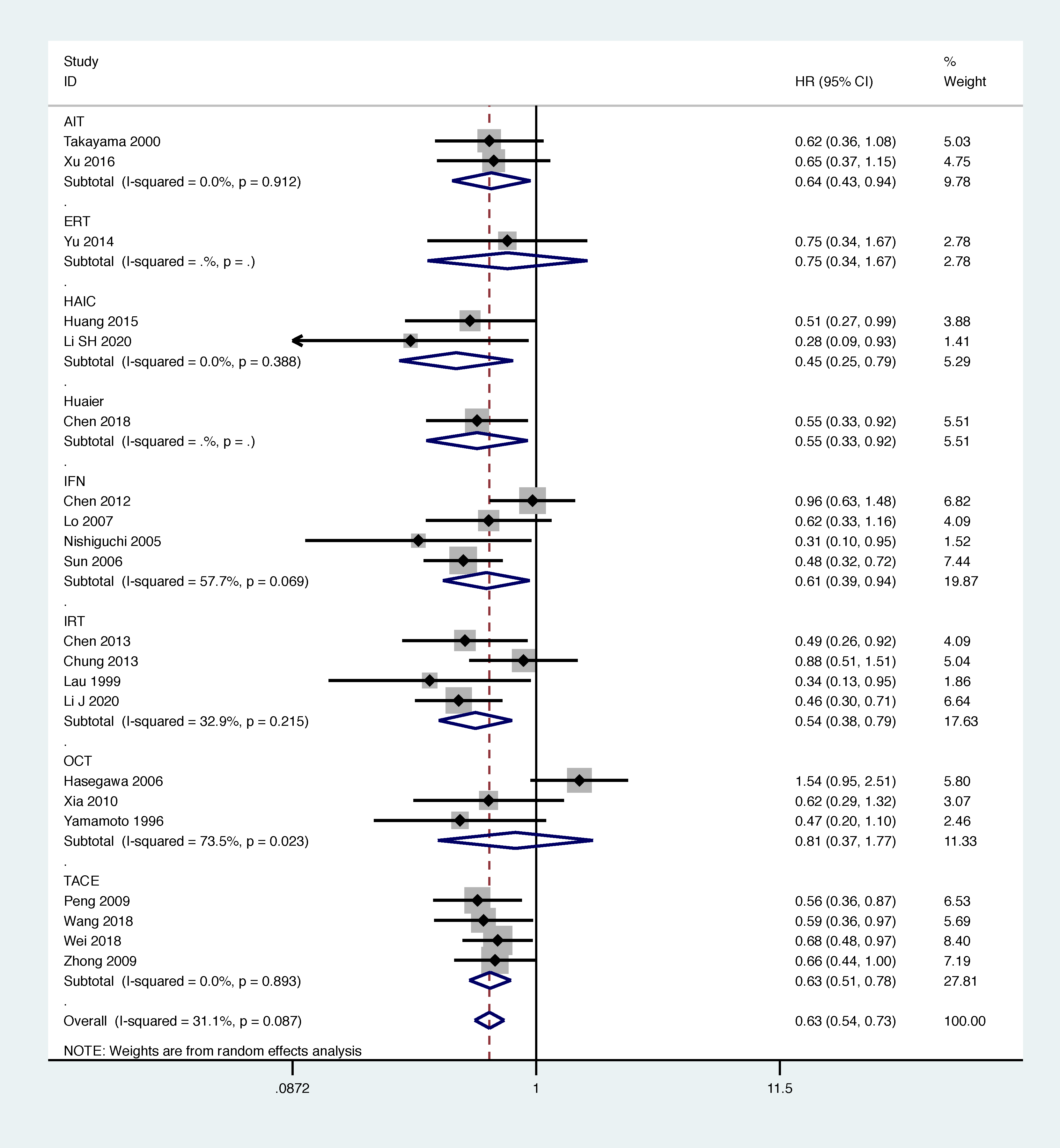


Supplementary Figure 4. Network diagram of eligible comparisons for recurrence. Each circular node represents a variety of interventions. The circle size is proportional to the number of randomly assigned participants. The width of lines between the nodes is proportional to the number of trials performing head-to-head comparisons.


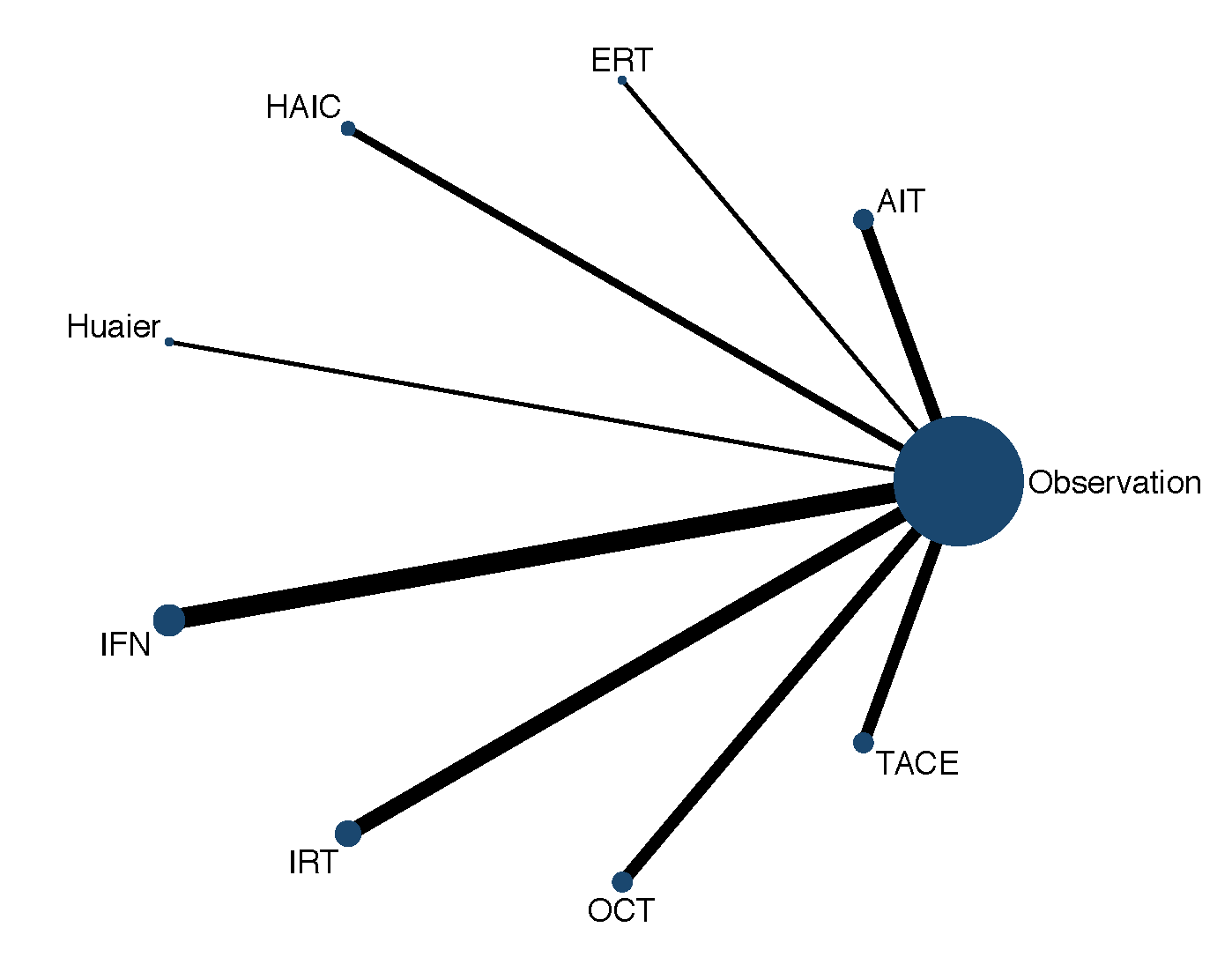


Supplementary Figure 5. Funnel plot for the efficacy of adjuvant treatments on preventing recurrence (A) and contributing OS (B).


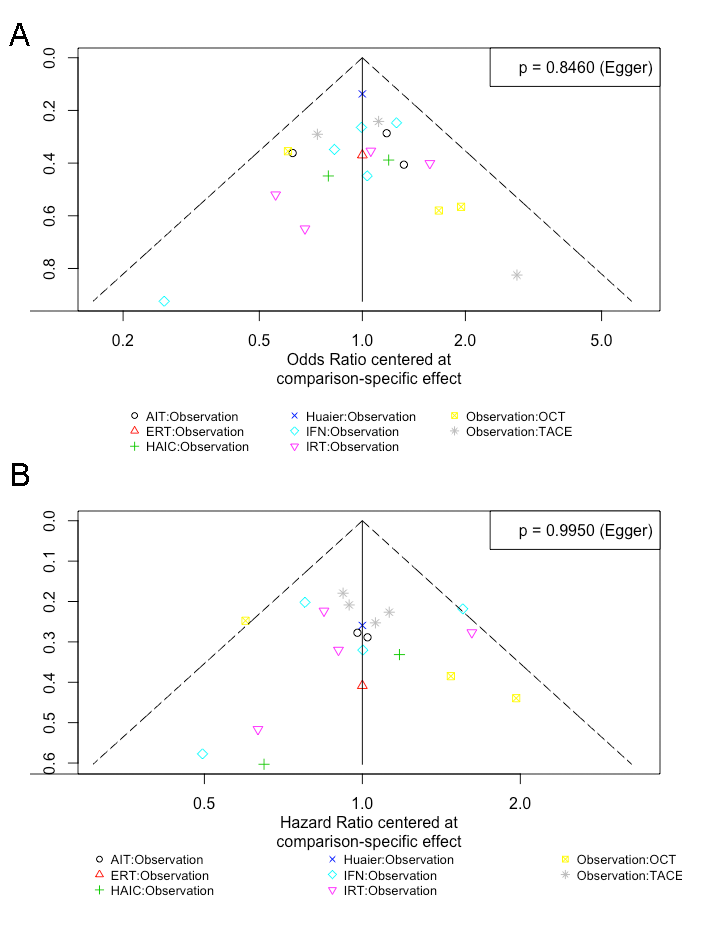

Supplement: Supplementary file 1 [file DataSheet_1.docx]
